# Granzymes: The Molecular Executors of Immune-Mediated Cytotoxicity

**DOI:** 10.3390/ijms23031833

**Published:** 2022-02-06

**Authors:** Zachary L. Z. Hay, Jill E. Slansky

**Affiliations:** Department of Immunology and Microbiology, Anschutz Medical Campus, University of Colorado School of Medicine, Aurora, CO 80045, USA; zachary.hay@cuanschutz.edu

**Keywords:** granzyme, cytotoxicity, T cell, cell death, immunogenic cell death, tumor-infiltrating lymphocytes

## Abstract

Cytotoxic T lymphocytes, differentiated CD8+ T cells, use multiple mechanisms to mediate their function, including release of granules containing perforin and granzymes at target cells. Granzymes are a family of cytotoxic proteases that each act on unique sets of biological substrates within target cells, usually to induce cell death. Granzymes are differentially expressed within T cells, depending on their environment and activation state, making the granzyme cytotoxic pathway dynamic and responsive to individual circumstances. In this review, we describe what is currently known about granzyme structure, processing, and granzyme-induced cell death in the context of cancer and in some other inflammatory diseases.

## 1. Introduction

Granzymes (Gzms), a family of cytotoxic proteases, are key players in cytotoxic T lymphocyte (CTL) elimination of target cells. Gzms are also expressed by other immune cells, such as natural killer cells [1,2] and mast cells [3], but we mainly focus on CD8 T cell expression of Gzms here. Activated CD8 T cells recognize and eliminate their targets as their T cell receptor (TCR) interacts with target cell MHC class I molecules loaded with peptide (pMHC). The T cells’ environment allows dynamic T cell responses to pMHC. This dynamic response includes elimination of target cells through expression of specific Gzms. A more complete understanding of the Gzm system may reveal potential molecular targets to improve CTL activity against cancer and other cells.

Recent breakthroughs utilizing the human immune system to target and eliminate malignancies has revolutionized how cancer is treated. Checkpoint blockade therapy, adoptive T cell therapy, and chimeric antigen receptor T cell therapy have shown clinical benefits and rely on generating or augmenting a T cell response against cancer cells for their efficacy. T cells mediate their cytotoxic function through multiple mechanisms, which is of particular interest given the advancements in immunotherapies [4,5]. While T cells have a multitude of functions in regulating their microenvironments, such as modulating angiogenesis [6], affecting the inflammatory state [7], and recruiting other immune cells [8], a critical function remains—killing of target cells in an antigen-specific manner. Elimination of specific target cells is a key facet of the effectiveness of cancer immunotherapies. Targeting the cytotoxic activities of T cells may prove critical to improving the efficacy of treatments without increasing unwanted side effects or increasing the number of T cells needed for treatment.

There are three main pathways that CTLs use to kill target cells: the death ligand pathway, production of cytokine interferon gamma (IFNγ), and the cytotoxic granule pathway. In the death ligand pathway, a secreted or cell surface ligand, such as Fas ligand (FasL) or TNF-related apoptosis inducing ligand (TRAIL), interacts with its receptor on the target cell, which induces cell death. The Fas/FasL interaction leads to activation of the caspase cascade and apoptosis in target cells ensues [9,10,11]. This pathway relies on target cell expression of receptors for death ligands, which are modulated by target tumor cells, preventing activation of cell death, and providing a mechanism of escape from immune-mediated cytotoxicity [12].

IFNγ reduces tumor cell proliferation through activation of the transcription factor STAT1, which regulates the kinase inhibitor p21 and inhibits cell cycle progression [13]. IFNγ also increases tumor cell death by increasing the expression of Fas and TRAIL [14,15] and potentiates increased apoptosis by upregulating the expression of multiple caspases [16,17]. Finally, IFNγ directly acts on endothelial cells to stop blood flow in vascularized tumors leading to tumor collapse [18]. While much of IFNγ’s activity is indirectly cytotoxic, relying on other mechanisms for ultimate destruction of tumor cells, it has a critical role in T cell-mediated tumor cell lysis.

CTLs primarily eliminate target tumor cells using cytotoxic granules [19]. CTLs store cytotoxic molecules in specialized lysosomes/secretory vesicles termed granules that, upon ligation of their T cell receptor (TCR), are released towards the immunological synapse at the target cell [20,21]. In addition to Gzms, cytotoxic granules contain a host of other proteases, including cathepsins, which can activate other lysosomal proteases [22] and granulysin, which can destroy intracellular microbial membranes [23,24,25]. Cytotoxic granules also contain perforin, a pore-forming protein that damages the cellular membrane of target cells and mediates entry of Gzms into the target cell cytosol [26,27,28]. The Gzm proteases each act upon specific and unique substrates within the target cell to initiate cell death [29,30,31].

Gzms are critical to multiple disease states, contributing to the progression of atopic dermatitis [32,33], cardiovascular disease [34], diabetes [35], and other inflammatory diseases, including sepsis [36]. Gzm B inhibition also reduces disease severity in autoimmune skin blistering diseases [37]. Increased expression of Gzms is therefore not inherently protective; in fact, it participates in a range of pathologies. The disease processes that Gzms contribute to highlight their destructive potential, necessary for immune-mediated defense against intracellular pathogens and malignancies, but they are also a danger when dysregulated. The cytotoxic activity requires regulation of Gzm production critical to the effective, but safe, functioning of cytotoxic immune cells. The contribution of Gzms to these different disease states may make them ideal targets to treat a range of immune-mediated diseases and their participation in protective immune responses could be key to enhancing targeted cytotoxicity against cancer cells.

## 2. Granzymes

The Gzm enzymes can be divided into three subclasses separated by chromosomal location (Table 1). The Gzm A locus of Gzms contains Gzm A and K and is located on chromosome 13 in mice and chromosome 5 in humans [38,39]. The Gzm B locus that contains Gzm B, and a varying number of related Gzms, depending on the organism, is contained on chromosome 14 in both mouse and human genomes [39,40]. The Gzm M locus, which contains only Gzm M, is located on chromosome 10 in mice and 19 in humans [39].

The Gzm B locus contains Gzm B and Gzm H in humans, and in mice contains Gzm C–G and N. The mouse Gzm B locus is organized as (5′ → 3′) Gzm B, C, F, N, G, D, E. All Gzms from this locus other than Gzm B are orphan Gzms, designating that they do not yet have defined substrates. Little is currently known about these orphan Gzms in either humans or mice; however, experiments using recombinant Gzm proteins have demonstrated the capacity of these molecules to induce unique forms of cell death in target cancer cells and unique expression profiles of cytotoxic mediators, even those contained within the same locus. Experimental immunotherapies in the early phases of development are testing different death pathways to improve the immune response against cancer [41,42,43,44,45].

## 3. Gzm Sequence and Phylogeny Analysis

**Figure 1:**
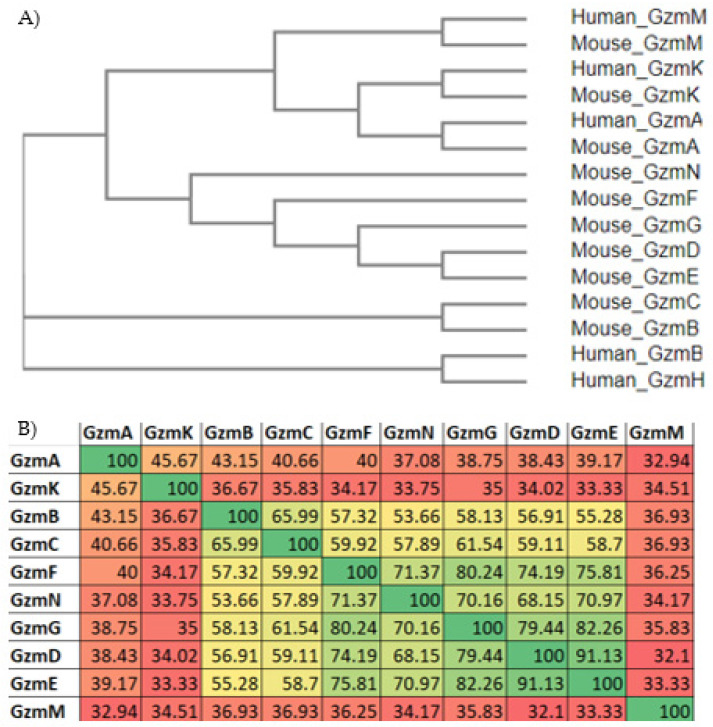
Gzm sequence homology and predicted relationships within this class of proteases. Alignments generated using Clustal Omega [.tar.gz (1.2.4)]. (**A**) The dendrogram depicts predicted phylogenetic relationships based on protein sequences of all mouse and human Gzms. (**B**) This table shows the percentage each mouse Gzm is homologous with every other mouse Gzm; similarity increases as the color trend green and decreases as the color tends red.

Phylogenetic trees constructed from the protein sequences encoding each Gzm in humans and mice, which show similarity between Gzms A, K, and M, suggesting that they likely evolved together and that these Gzms predate the divergence of rodents and primates (Figure 1A). Gzm H in humans and Gzm C in mice most closely resemble human and mouse Gzm B, respectively. Additionally, both Gzms are adjacent to their respective Gzm B suggesting similarity of function between Gzms H and C. However, phylogenetic analysis does not predict the substrate specificity of each individual Gzm, even among largely homologous enzymes. The potential divergence of target substrates between species adds additional complexity to comparing enzyme activities between species. The remaining Gzms in the mouse Gzm B locus all cluster with one another, and many have the most sequence homology of all mouse Gzms (Figure 1B) but remain the least studied Gzms. The greatest sequence homology within Gzms is found on the same locus. Gzm M is the sole member of its locus and has the least sequence homology with all other Gzms, sharing no more than 36.9% of its sequence with any other Gzm. Gzms D and E share the greatest homology with 91.1% shared sequence.

The presence of additional Gzm genes in the mouse genome suggests that mice had more pressure to maintain a greater range or redundancy in immune-mediated cytotoxicity to combat evasive intracellular infections, and that some mouse Gzms may function in response to specific pathogens or cell death evasion tactics. The lack of homologous Gzms for all mouse Gzms does not preclude the possibility that these orphan mouse Gzms could retain functionality in human cells and may retain their specific cytotoxic function across species, which has been confirmed in multiple Gzms already [46]. Alternatively, other yet-unidentified molecules in human cells may replace the function of these mouse Gzms.

**Figure 2:**
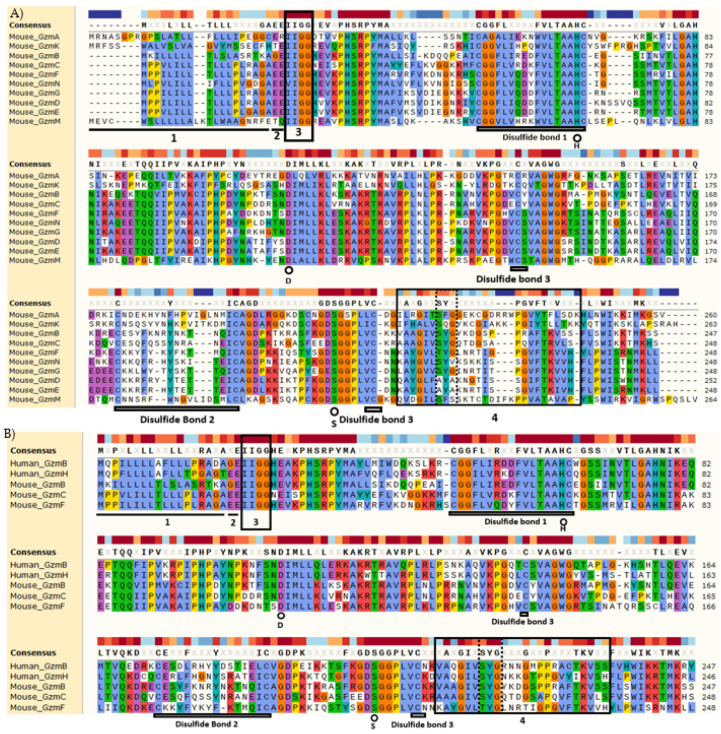
Protein sequence alignment of selected Gzms. Alignments were generated using Snapgene’s MUSCLE alignment and ordered based on species and chromosomal location. A consensus sequence, shown in black above each individual Gzm sequence, is given when there was 60% or greater consensus amongst Gzms examined. Comparing select Gzms from the human and mouse Gzm B locus (**A**) or comparing all mouse Gzms (**B**), there is considerable similarity between each Gzm sequence examined, but the differences observed change the substrates of individual Gzm and their cytotoxic activity. Annotations in both (**A**,**B**) mark the same features. Line 1 indicates the leader sequence and line 2 indicates the dipeptide that is cleaved to fully activate each individual Gzm. The box marked 3 is the start of the active enzyme, and this “IIGG” sequence is highly conserved amongst all Gzm. Highly conserved disulfide bonds are marked with a double line beneath the cystine residues that form the bonds, and stretching between cystine residues, that comprise the bonds where possible. These disulfide bonds form variable loops that are critical to the structure and function of each Gzm. The three highly conserved residues making up the catalytic triad are marked with a black circle above the conversed residues symbol, histidine (H), aspartic acid (D), and serine (S). The catalytic triad participates in the active site of all Gzms, which is located at the junction of two beta-sheet domains. Box 4 indicates the substrate binding subsite 1, which contains a critical functional motif for substrate binding specificity shown within the dotted box. The Gzm B locus Gzms have a conserved tyrosine (Y) as the central residue in this motif, whereas other Gzms have less restriction.

While each Gzm has been shown to act on unique sets of substrates, thus far (Table 2), and even have divergent cytotoxic functions, they remain largely homologous in many ways. All Gzms share critically conserved amino acid sequence elements that confer similarities in their enzymatic activities (Figure 2A,B). By aligning Gzm sequences, we determined the percent sequence homology shared between all Gzms (Figure 1B).

All Gzms are produced as zymogens, or proenzymes, that require both the cleavage of a leader sequence and a dipeptide to become fully activated [69,70,71]. The leader sequence is predominantly hydrophobic residues in all Gzms, although the sequence and sequence length diverge somewhat, especially in the Gzm B locus (Figure 2). The dipeptide is largely conserved amongst Gzms, especially within the Gzm B locus, where they all share the same dipeptide amino acid sequence “EE,” except for Gzm B itself. Each active Gzm starts with the highly conserved “IIGG” amino acids, which is present in all Gzms except Gzm N. Each Gzm contains three sets of cystine residues that form disulfide bonds which dictate the Gzm structure. Marked with small circles in Figure 2A,B, there are the highly conserved residues that form the catalytic triad (S-H-D) in the active site of these Gzms. The S-H-D motif is one of the most common catalytic triads and relies on the histidine to polarize the serine hydroxyl group. This motif creates an effective nucleophile which initiates the enzymatic activity on substrates and is further characterized by the formation of an oxyanion hole which stabilizes charge buildup on substrate intermediates [72,73].

## 4. Gzm Production and Regulation

**Figure 3:**
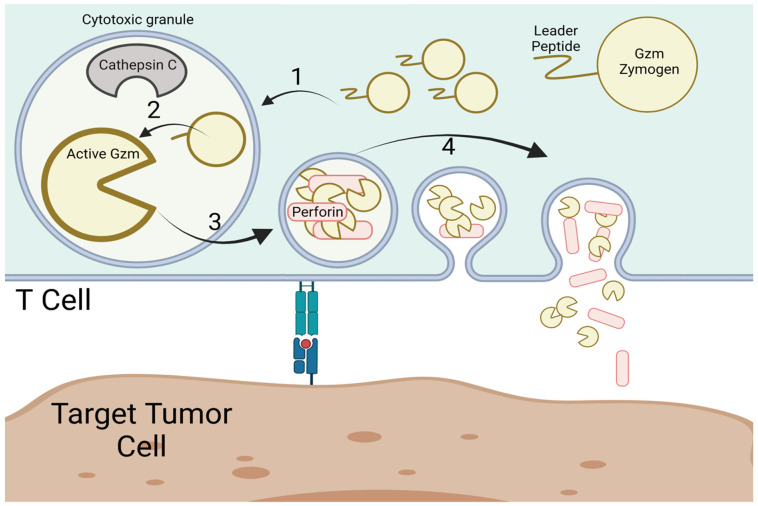
Gzm processing in cytotoxic T cells. Gzms are produced as zymogens with a leader sequence and dipeptide on the amino-terminus, which holds the Gzms in an enzymatically inactive conformation. (1) Gzms are transported and stored cytotoxic granules and their leader sequence is cleaved leaving behind a dipeptide. (2) In the cytotoxic granule cathepsins, a class of proteases present in lysosomes and cytotoxic granules, cleave the N-terminal dipeptide from the Gzms, converting them to their active conformation. (3) Gzms are stored in their active conformation in the cytotoxic granules until their release along with perforin, which mediates entry of Gzms through the target cell membrane into target cell cytosol. (4) Upon TCR activation the cytotoxic granules are released towards the immunologic synapse. Perforin mediates entry of Gzms into the cytosol of target cells where Gzms can initiate they cytotoxic activity.

Given the cytotoxic potential and potent effects on immunity that many Gzms have, CTLs have evolved multiple methods to protect themselves from their own cytotoxic proteases. CTLs are safeguarded by producing Gzms in an inactive state. As discussed above, a highly conserved feature of all Gzms, across species, is their production as an inactive zymogen [69,70,71]. Gzms are translated with both a leader sequence and a dipeptide that must be cleaved from the N-terminus before the Gzms are enzymatically active [69,70,71] (Figure 3). The leader sequence is predicted to mediate entry into the cytotoxic granule and is cleaved in the process, though the Gzm remains inactive due to the dipeptide sequence. Cathepsin C and other proteases stored in the granules cleave the dipeptide [74]. The active Gzms are stored safely inside the granules, segregated from the cytosol, to avoid damage to the CTL in which they were produced.

In addition to segregation and storage of Gzms within CTL, transcription of Gzm mRNA is also regulated in different environments and activation states of the CTL. In fact, Gzms are some of the most differentially regulated genes in T cells, with some of the orphan Gzms having the most distinct differences in expression based upon the environment in which the T cell is found [75]. Further elucidation of the function of each of these Gzms is needed to conclude if the orphan Gzms are playing a critical role through their unique substrate specificity and function in mediating an effective immune response.

While there are multiple steps between mRNA production and production of the active Gzm, these steps do not appear to be mechanisms regulating the expression of these proteins, just their activity within the cells in which they are produced. RNA expression strongly correlates with protein expression for Gzms in T cells and suggests that Gzm production is largely regulated at the RNA transcript level, rather than post-transcriptionally [76,77].

**Figure 4:**
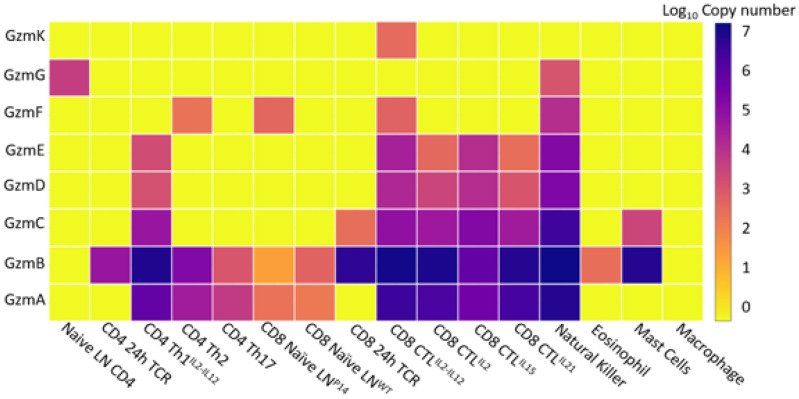
Gzm protein expression within hematopoietic cells as determined by mass spectrometry. Using the Immunological proteomic resource (ImmPRes), an open access proteomic database of murine immune cell populations generated using large scale quantitative mass-spectrometry, we examined Gzm expression within hematopoietic cells. Identification of protein expression utilized data-dependent acquisition tandem mass spectrometry. Copy number refers to the average protein concentration per cell. From this dataset we have found that GzmB seems to have the most diverse expression within different cell types examined and is often the most abundant Gzm. Other Gzms have more unique expression patterns and are only expressed on a smaller subset of the immune cells examined. Critically, T cell expression of most Gzms seems to be dependent on their activation status.

Gzms can be expressed by multiple different immune cell subtypes and the expression pattern of each Gzm within different cell subtypes differs. Using the Immunological Proteomic Resource (ImmPRes), we determined the amount Gzm proteins produced in different immune cell subtypes in mice hematopoietic cells (Figure 4). The hematopoietic cells probed in the ImmPRes dataset include T cells, natural killer cells, eosinophils, mast cells, and macrophages, and is a powerful resource to determine protein production of difficult to measure molecules, including the majority of the Gzms, which do not yet have a commercial antibody available for detection. ImmPRes probed T cells from different lineages and in multiple conditions. From this dataset we show that each Gzm is differentially regulated in T cells after alternative forms of activation. However, this dataset does not represent every environmental pressure that affects T cells and their production of Gzms. Within the tumor microenvironment (TME), multiple Gzms are increased in CD8+ T cells [78,79], indicating that Gzm expression is responsive to T cell environmental pressures, not all of which can be measured in vitro.

Gzm B is expressed in the widest range of immune cells, including every T cell subset examined apart from naive CD4+ T cells and macrophages. Gzm B also appears to be the most abundant Gzm in most of the cell populations in which Gzms are expressed, followed by GzmA. The increased abundance of Gzm A and B over other Gzms in ImmPRes is consistent with current literature, which has long suggested that these Gzms are the most abundant [80,81].

Differences in Gzm expression between CTLs from different environments and activation states has driven research preferentially into studying Gzms highly expressed in the models most commonly available, and has likely precluded investigation into Gzms expressed in more specific T cell subsets. These more restrictively expressed Gzms may represent more effective cytotoxic molecules that are only upregulated upon specific environmental cues or stimulation events and may warrant their further investigation.

The requirement for expression of not only a specific Gzm in the immune cell but for its unique substrate to be present in the target cell adds further difficulty to understanding the complex interactions needed for Gzm-induced cell death.

## 5. Cytotoxicity and Other Impacts of Gzms

Not all Gzms have specific cytotoxic functions, and some are non-cytotoxic intra and extracellularly [82,83,84]. However, the main function of most Gzms is still presumed to be direct elimination of target cells, though the specific mechanism and type of cell death induced varies. Using Gzms as a tool to improve the effector function of T cells may increase the efficacy of cancer immunotherapies, but to test this possibility, further understanding of each Gzm’s molecular function is still required.

**Figure 5:**
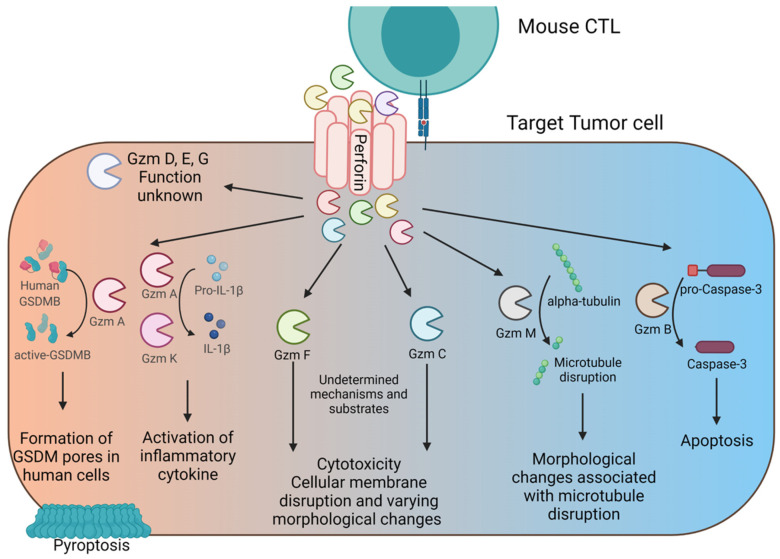
The different pathways of Gzm-mediated cytotoxicity. CTL degranulate towards the immunological synapse, releasing perforin and Gzms. Perforin forms pores in target cell membranes and facilities Gzm entry into the cytosol of target cells. Once in the cytosol each Gzm acts upon unique substrates to initiate their divergent functions. Gzm A and K have been shown to be largely inflammatory, and their primary function may be to induce release of inflammatory cytokines rather than directly inducing cell death. Gzm F is shown to induce rupture of the cellular membrane through an unknown mechanism that closely resembles necroptotic cell death. Gzm C also induces rupture of the cell membrane through an unknown mechanism and induces other unique cytotoxic changes. Gzm M can act directly on microtubule substrates. Gzm B induces apoptosis by acting directly on caspases. Gzms can be produced simultaneously by the same cell, but many of them are highly transcriptionally regulated in the T cell, so multiple of these pathways can be activated at the same time, depending on the T cells specific expression of each Gzm.

Below we will describe what is currently known about the function of individual Gzms, which is summarized in Table 2 and in Figure 5.

### 5.1. Gzm B

Gzm B is the best studied and characterized Gzm, and as such there are many reagents available for further study. Gzm B knock out mice [85], colorimetric assays to measure Gzm B enzymatic activity [86], recombinant Gzm B proteins [87,88], multiple antibody clones against human and mice Gzm B to allow for detection by flow cytometry, Western blot, immune histochemistry and ELISA [22,89] have all facilitated experiments to understand Gzm B better than the other Gzms.

Gzm B activates the caspase cascade through cleavage of one of the primary executioners of apoptosis, caspase-3, and in humans specifically, through the cleavage of the protein Bid, which is also a part of the canonical apoptotic pathway [90,91,92,93,94] (Figure 5). This process culminates in cell blebbing, where cellular contents are packaged and released, followed by efficient phagocytosis of the dying cell before complete loss of cell membrane integrity [95]. Apoptosis is a largely “immunologically silent” or even suppressive form of cell death [96,97,98]. Gzm B is one of the most widely expressed Gzms in T cells regardless of activation status, whereas other Gzms are expressed in higher quantities under specific conditions [99]. This widespread expression of Gzm B highlights the importance that it induces a less immunogenic form of cell death and is likely an adaptive feature of Gzm B’s induction of canonical apoptotic cell death, rather than inducing a form of cell death that could increase inflammation.

Additionally, human Gzm B can cleave and activate Bid, into truncated Bid, which can then activate the proteins Bax and/or Bak. Bax and Bak oligomerize in the mitochondrial outer membrane, permeabilizing the membrane and allowing for the release of cytochrome C, which is usually stored in the mitochondrial inter-membrane space, into the cytosol [46]. Release of cytochrome C initiates the assembly of the apoptosome, composed of Apaf-1 and caspase-9, which is critical in activating other caspases and continuing the caspase cascade to induce apoptosis [100,101,102,103].

Mouse GzmB and Human GzmB both show enzymatic activity against caspase-3 and caspase-7, and both enzymes induce cell death in tumor cells from both species. However, their substrate preferences and activities diverge [46]. Human Gzm B alone cleaves BID from either species but can cleave only human ICAD and caspase-8. Mouse Gzm B does not cleave BID, ICAD, or caspase-8 from either species [46]. This highlights the specificity of Gzms for their substrates and how alterations between species in either the Gzm or the substrate can result in differences in activity. Ultimately, both human and mouse Gzm B have some shared substrates and are functional in both species to initiate the caspase cascade and trigger specifically apoptotic cell death (Table 2).

### 5.2. Gzm A

Early data showed that Gzm A and Gzm B are the most abundant Gzms and were the only Gzms detected, though at lower frequencies, in inactivated T cell subsets [80,81]. However, while other Gzms are not as widely expressed, their production can be drastically increased in smaller CTL subsets within specific environments and potentially different types of activation [75,78,79], and may represent important subpopulations of highly functional, or dysfunctional, T cells.

Gzm A function is not completely defined, and contradictory results are reported on the mechanism of cell death Gzm A induces [47,50,51,104]. However, multiple experiments show that Gzm A acts as an initiator of pro-inflammatory signaling within target cells [104,105]. More specifically, it is established that Gzm A directly cleaves a key mediator of the inflammatory response, the pro-cytokine IL-1B [50], which is necessary in the activation and secretion of this cytokine.

Additionally, Gzm A directly cleaves human gasdermin B, a member of the gasdermin family of proteins that forms pores in the plasma membrane [47]. Specifically, when Gzm A cleaves the gasdermin inhibitory domains the remaining pore forming domains oligomerize and bind to phospholipids of the plasma membrane, leaving a hole. Cell death ensues by pyroptosis [106,107,108], a pro-inflammatory form of cell death. Canonical pyroptosis was previously considered reliant on the formation of an inflammasome by activated caspase-1 and cleavage of gasdermin D [109,110]. However, gasdermin B is one of multiple gasdermins that are now known to be executioners of pyroptotic cell death [111,112]. Gzm A-induced cell death is caspase-independent [47,51,104], but through direct activation of gasdermin B, initiates the terminal step of the pyroptotic pathway.

Another substrate of Gzm A is the protein NDUFS3, which is a mitochondrial matrix protein and critical component of the electron transport chain [51]. Gzm A disruption of this process causes an increase in reactive oxygen species. This process is independent of mitochondrial outer membrane permeabilization (MOMP), does not result in the cleavage of Bid or release of apoptogenic factors [113], nor does it require bak or bax activity [114]. This unique combination of events suggests another mechanism through which Gzm A induces cell death: through an MOMP- and caspase-independent pathway, that generates reactive oxygen species and disrupts ATP generation.

While there are multiple different pathways Gzm A could be interacting with in target cells, the consensus amongst these studies is that Gzm A functions to induce an inflammatory form cytotoxicity.

### 5.3. Gzm M

Gzm M is the only member of the Gzm M locus [39] and can induce cytotoxicity in target human cells though not target mouse cells [67]. However, the exact nature of this cell death, if it is observed, varies between studies [64,67]. Gzm M has been shown to induce both a caspase-dependent and a caspase-independent form of apoptotic cell death in humans [64,115]. Gzm M is largely expressed by natural killer cells, though CTLs also express this enzyme. One mechanism through which human Gzm M executes its cytotoxic function is through cleavage of the protein Fas-associated protein with death domain (FADD) [64]. Cleaved FADD self-oligomerizes and associates with caspase-8, which is then processed into its active state to initiate the caspase cascade [116]. While both mouse and human Gzm M induce apoptosis in human cancer cells in vitro, Gzm M from either species does not induce apoptosis in mouse cancer cells [67]. Other studies report that natural killer cells from Gzm M-deficient mice have impaired control of tumor growth in vivo [117], suggesting a critical function of mouse Gzm M outside of direct induction of apoptosis. Gzm M-induced cell death induces morphological changes in target cells, including chromatin condensation and the presence of large cytoplasmic vacuoles. Both mouse and human Gzm M cleave α-tubulin of either species and disrupt the microtubule network [67], contributing to observed morphological changes [118]. These studies present mechanisms through which both mouse and human Gzm M affect target cancer cells; although, they also highlight how changes in Gzm substrates between species can impact the function of individual Gzms.

### 5.4. Gzm K

Gzm K is clustered and most homologous with Gzm A. Gzm K and A share some substrate specificities, though Gzm K does have other unique functions. In humans, Gzm K has been found to have multiple biological substrates, including SET [59], Bid [119], and Ape1 [62]. Critically, Gzm A does not have enzymatic activity against Bid, highlighting a potential functional difference between these Gzms. In mice, the function of Gzm K is less well studied, but similar to Gzm A, there is some evidence that mouse Gzm K initiates inflammation in target cells [120].

### 5.5. Gzm F

An initial study of Gzm F, an orphan mouse Gzm, suggests that it induces a caspase-independent, necroptotic-like form of cell death [55]. Necroptotic cell death is a regulated form of cell death that has strong immunogenic effects [121] in part through the release of cellular contents and damage-associated molecular patterns [122].

The induction of this immunogenic form of cell death in an antigen-specific manner could present a leverageable tool to influence the TME but requires a better understanding of the mechanism of Gzm F-induced cell death. Gzm F-induced cell death results in cellular membrane disruption and phosphatidylserine externalization at concentrations and time points where Gzm B induces phosphatidylserine externalization but no membrane disruption. These differences suggest a unique form of cell death induction. Additionally, Gzm F induces mitochondrial damage, and single-stranded DNA nicking, rather than DNA fragmentation, cellular organelle damage, and extensive vacuolization of the cytoplasm, indicative of necroptosis-like cell death [56]. Disruption of the mitochondria results in impairment of electron transport and ATP generation. ATP decline switches the form of cell death from apoptosis to more necrotic-like forms of cell death [57], demonstrating how Gzm F suppresses apoptosis by preventing the formation of the apoptosome. Gzm F impairment has a significant impact on CTL-induced cytotoxicity in caspase-inhibited tumor cells. Taken together, these findings suggest an immunogenic form of cell death unique amongst the Gzm family.

Analysis of publicly available datasets has shown that Gzm F is one of the most highly upregulated genes in CD8 T cells in the TME [78,79]. This increase in Gzm F could be a marker of a specific T cell activation status intended to increase cytotoxic activity in exhausted CD8 T cells or could be a mechanism for terminally exhausted T cells to eliminate themselves. Further work is still required to understand the function of one of the most highly upregulated genes in T cells within the TME and to determine the capacity of Gzm F-mediated necroptotic-like cell death to modulate the TME’s immunogenicity.

### 5.6. Gzm C

Little is currently known about Gzm C, an orphan mouse Gzm. However, preliminary studies of this protease have indicated that it induces cell death in target cells. Gzm C is directly downstream of Gzm B and has the greatest sequence homology to mouse Gzm B of all mouse Gzms, though the form of cytotoxicity is unique from Gzm B-induced cell death.

Gzm C-induced cell death is caspase-independent, and does not rely on activation of BID, or CAD nuclease, and results in rapid phosphatidylserine externalization and single-stranded DNA nicking, rather than double-stranded DNA cleavage [54]. Additionally, the molar quantity required for activity and the kinetics of Gzm C-induced cytotoxicity are similar to those for Gzm B-induced cell death, suggesting that Gzm C expression can influence the type of cell death induced and that it plays a non-redundant role in T cell-mediated cytotoxicity [54].

### 5.7. Gzm H

Gzm H is the only other granzyme located on the Gzm B locus in humans. Gzm H is still poorly understood, though there has been some preliminary work to define Gzm H’s function. In one study, Gzm H acted independently of caspases, did not activate Bid, and did not induce the release of cytochrome C [123], suggesting that Gzm H induces a non-apoptotic form of cell death. However, another study found Gzm H induces a caspase-dependent form of cell death that directly activates Bid and induces DNA fragmentation, similar to Gzm B activity [124]. Both studies used recombinant Gzm H but came to conflicting conclusions regarding the mechanism of Gzm H-induced cell death. Critically, both agreed that Gzm H has cytotoxic activities.

## 6. Conclusions

Gzms are often released in the terminal step of T cell interactions with target cells and present a means for antigen-specific T cells to directly influence the TME. The specific pathway each Gzm impacts target cells is being elucidated; although, many Gzms remain poorly understood. Taken together, the lack of fully defined pathways and potential for direct, antigen-specific, immune-mediated manipulation of the TME make the Gzm molecules prime targets for further investigation and potential immunotherapies.

The granule exocytosis pathway participates in a wide range of cellular mechanisms to induce cytotoxicity in target cells. While we have described what is known about the function of Gzms in CTLs individually, Gzms are not expressed individually. One CTL expresses multiple different Gzm genes at different levels under different conditions [99]. Thus, any crosstalk between Gzms and the microenvironment or each other may have consequences. The studies to date of individual Gzms may not reveal the full picture. Each Gzm works in concert with other Gzms, activating different pathways simultaneously, and any synergistic effects will depend on the array of Gzms being produced by a given CTL. While research to examine individual Gzms is critical to furthering our understanding, experiments examining how these Gzms work in collaboration, or in genetically engineered immune cells directly, are notably lacking from the field at present.

Studies using mass cytometry have confirmed that separate T cell differentiation stages utilize different and specialized cytotoxic programs, as characterized by their expression of different Gzms and cytotoxic mediators measured [99]. The cytotoxic profile may contribute to predicting patient outcomes, design more effective immunotherapies, and suggest the importance of understanding when and where each CTL expresses individual Gzms.

Gzms are some of the most differentially expressed genes in T cells taken from different environments [75,78,79]. Gzm B is often used to gauge T cell activation and cytotoxic capacity. However, other Gzms have more significant changes in their expression levels than Gzm B, suggesting that Gzm B expression alone is not sufficient to predict T cell function. The alternative functions of the whole Gzm family indicates that CTL could be using the mechanism of cell death to further orchestrate and control the immune response subsequent to target cell destruction. Alternative forms of cell death may impact the TME, stimulating or suppressing immunogenicity. This dynamic may be a component of T cell-mediated cancer treatment that is not yet effectively leveraged. Gzms are the key mechanism utilized by CTLs to directly eliminate cancer cells, so furthering our understanding of the functions of each Gzm represents an unmet opportunity to improve T cell-mediated immunotherapies. 

## Figures and Tables

**Table 1:** Granzyme chromosomal location and size in mice (A) and humans (B) ^1^.

(A)			
	Chromosome	Location	Length within Genome (bp)
**Gzm K**	13 c	chr13:113,308,172-113,317,431	9260
**Gzm A**	13 c	chr13:113,230,361-113,237,515	7155
**Gzm B**	14 c	chr14:56,496,295-56,499,717	3423
**Gzm C**	14 c	chr14:56,468,898-56,472,113	3216
**Gzm F**	14 c	chr14:56,442,720-56,448,874	6155
**Gzm N**	14 c	chr14:56,403,254-56,412,056	8803
**Gzm G**	14 c	chr14:56,394,039-56,397,036	2998
**Gzm D**	14 c	chr14:56,367,013-56,370,065	3053
**Gzm E**	14 c	chr14:56,355,083-56,358,082	3000
**Gzm M**	10 c	chr10:79,524,854-79,531,095	6242
**(B)**			
	**Chromosome**	**Location**	**Length within Genome (bp)**
**Gzm K**	5	chr5:55,024,256-55,034,570	10,315
**Gzm A**	5	chr5:55,102,646-55,110,252	7607
**Gzm B**	14 c	chr14:24,630,954-24,634,190	3237
**Gzm H**	14 c	chr14:24,606,480-24,609,685	3206
**Gzm M**	19 c	chr19:544,053-549,922	5870

^1^ Gzms are clustered on 3 different chromosomes, the Gzm A locus (blue), the Gzm B locus (orange), and the Gzm M locus (gray). Genome data was extracted from the UCSC genome browser using the Mouse Jun 2020 GRCm39/mm39 assembly and the Human Dec 2013 GRCh38/hg38 assembly. Gzms located on the complementary strand of the chromosome are identified with a “c” in the chromosome column.

**Table 2:** Substrates and functions of all mouse Gzms and human homologues if known ^1^.

	Known Substrates	Function
Gzm A *	Gasdermin B [47], SET [48,49], pro–IL-1β [50], NDUFS3 [51], histone H1 [52], HMGB2 [52], and ApeI [53]	Gzm A’s primary role is to activate and release pro-inflammatory cytokines from target cells. Mouse and human Gzm A induces pyroptosis in human cells through the cleavage and activation of human gasdermin B (there is no known mouse homologue for gasdermin B) [47]. Additionally, Gzm A can target multiple nuclear and mitochondrial targets that likely contribute to its specific form of caspase-independent cytotoxicity.
Gzm B *	Mouse and Human: pro-caspase 3, pro-caspase 7 Human only: Bid, inhibitor of caspase-activated DNase (ICAD), pro-caspase 8 [46]	Gzm B cell death results in the activation of the caspase cascade and induction of canonical apoptosis in both mouse and humans.
Gzm C	Orphan (undetermined)	Gzm C induces a caspase-independent form of cell death that results in single-stranded DNA nicking and mitochondrial swelling. Gzm C does not activate BID or the CAD nuclease, suggesting it has alternative targets and initiates different pathways from Gzm B [54].
Gzm D	Orphan (undetermined)	Undetermined
Gzm E	Orphan (undetermined)	Undetermined
Gzm F	Orphan (undetermined)	Gzm F has been suggested to induce a “necroptotic-like” form of cell death[55]. Gzm F-induced cell death is caspase-independent, induces mitochondrial damage independent of the Bid pathway, results in rupture of the cellular membrane, single stranded DNA nicking, cellular organelle damage, and extensive vacuolization of the cytoplasm [55]. This death phenotype strongly resembles necroptosis [56,57].
Gzm G	Orphan (undetermined)	Undetermined
Gzm K *	SET [58,59], β-tubulin [60], APE1 [59,61,62]	Gzm K induces single-stranded DNA damage, mitochondrial dysfunction, and generation of reactive oxygen species (ROS), and cell membrane damage through a caspase-independent mechanism [58,59].
Gzm M *	Human: nucleophosmin [63], FADD [64], survivin [65], ICAD [66] Mouse and Human: alpha-Tubulin [67]	Gzm M induces apoptosis in humans though its function is undetermined in mice. Gzm M does not induce cell death in multiple mouse tumor models in vitro but mouse Gzm M can induce apoptosis in target human cancer cells in vitro [67].
Gzm N	Orphan (undetermined)	To our knowledge, Gzm N has not been found to be significantly expressed in T cells or other immune cells. Its expression has been found in the testes in one study, specifically in spermatocytes and spermatids, though its biological function was not determined [68].

^1^ Gzms without a known substrate are designated orphan and mouse Gzms with a known human homologue with the same name are marked with an asterisk.

## Data Availability

Data sharing not applicable.
